# Vertical-to-Horizontal Rotational Myocutaneous Flap for Repairing Cicatricial Lower Lid Ectropion: A Novel Surgical Technique

**DOI:** 10.1155/2017/8614580

**Published:** 2017-10-18

**Authors:** Yu-Fan Chang, Chieh-Chih Tsai, Hui-Chuan Kau, Catherine Jui-Ling Liu

**Affiliations:** ^1^Department of Ophthalmology, Taipei Veterans General Hospital, Taipei, Taiwan; ^2^School of Medicine, National Yang-Ming University, Taipei, Taiwan; ^3^Department of Ophthalmology, Koo Foundation Sun Yat-Sen Cancer Center, Taipei, Taiwan

## Abstract

**Objective:**

To evaluate the efficacy and complications of a novel surgical technique for cicatricial lower lid ectropion that uses a vertical-to-horizontal (V-to-H) rotational myocutaneous flap procedure (Tsai procedure).

**Methods:**

We performed the V-to-H rotational myocutaneous flap procedure on 20 eyelids in 20 patients with mild to moderate cicatricial lower lid ectropion. A vertical myocutaneous flap was created from the anterior lamella of the vertical pedicle in the lateral third of the lower eyelid. Following a horizontal relaxing incision from the base of the flap, a vertical myocutaneous flap was created and rotated to horizontal. Two patients with combined cicatricial ectropion and paralytic lagophthalmos simultaneously underwent additional lateral tarsorrhaphy.

**Results:**

After a minimum follow-up period of 6 months, all patients showed good anatomical and functional improvement with decreased dependence on topical lubricants and a satisfactory cosmetic appearance. Two patients with combined cicatricial and paralytic ectropion had mild residual asymptomatic lagophthalmos. No patients required further revision surgery and there were no complications or recurrence.

**Conclusion:**

The V-to-H rotational myocutaneous flap technique was an effective and simple one-stage procedure for correcting cicatricial lower lid ectropion. It lengthened the anterior lamella and tightened horizontal eyelid laxity without the need for a free skin graft.

## 1. Introduction

Ectropion, an outward rotation of the eyelid margin, affects the lower eyelid and causes ocular irritation, conjunctival inflammation, and corneal exposure. Cicatricial lower lid ectropion is characterized by vertical shortening and/or scarring of the anterior lamella of eyelid and can be the result of thermal or chemical burns, mechanical or surgical trauma/scars, medications, sun damage, chronic inflammation, and involutional changes [1, 2]. Various techniques have been described for correcting cicatricial ectropion by lengthening the anterior lamella with transposition flaps or with full-thickness free skin grafts [3–11]. However, cicatricial ectropion is often accompanied by horizontal lid laxity due to involution changes; thus, both factors should be corrected to achieve a better outcome. Here we describe a novel surgical technique for lower lid cicatricial ectropion that uses a vertical-to-horizontal (V-to-H) rotational myocutaneous flap.

## 2. Methods

### 2.1. Patients

A retrospective medical record review was performed for all patients who underwent V-to-H rotational myocutaneous flap surgery for mild to moderate lower lid cicatricial ectropion with inferior sclera show (the distance of white sclera below the lower border of the corneal limbus) less than 8 mm at the Taipei Veterans General Hospital Eye Clinic from 2008 to 2016.  Table 1 showed detailed information of the 20 patients. All operations were performed by the same surgeon (CCT). The success of the outcome was assessed based on the anatomical restoration of the apposition of the lower lid to the globe, patient dependence on topical lubricants, and patient satisfaction with the cosmetic and functional results. Patient satisfaction with the cosmetic and functional outcomes was assessed 6 months after the operation as being satisfactory, acceptable, or unsatisfactory. An additional outcome assessment included flap viability and possible complications, including hematoma, infection, flap contracture, or the need for additional surgery.

### 2.2. Statistical Analysis

The Microsoft Excel 2010 statistical package was used to analyze the results, and data are presented as means ± standard deviations (SDs). The number of topical lubricants used before versus after surgery was compared using the paired *t*-test, with *P* < 0.05 considered statistically significant.

### 2.3. Surgical Technique

All procedures were performed under local anesthesia using 1% lidocaine plus adrenaline 1 : 100000.

**Figure 1:**
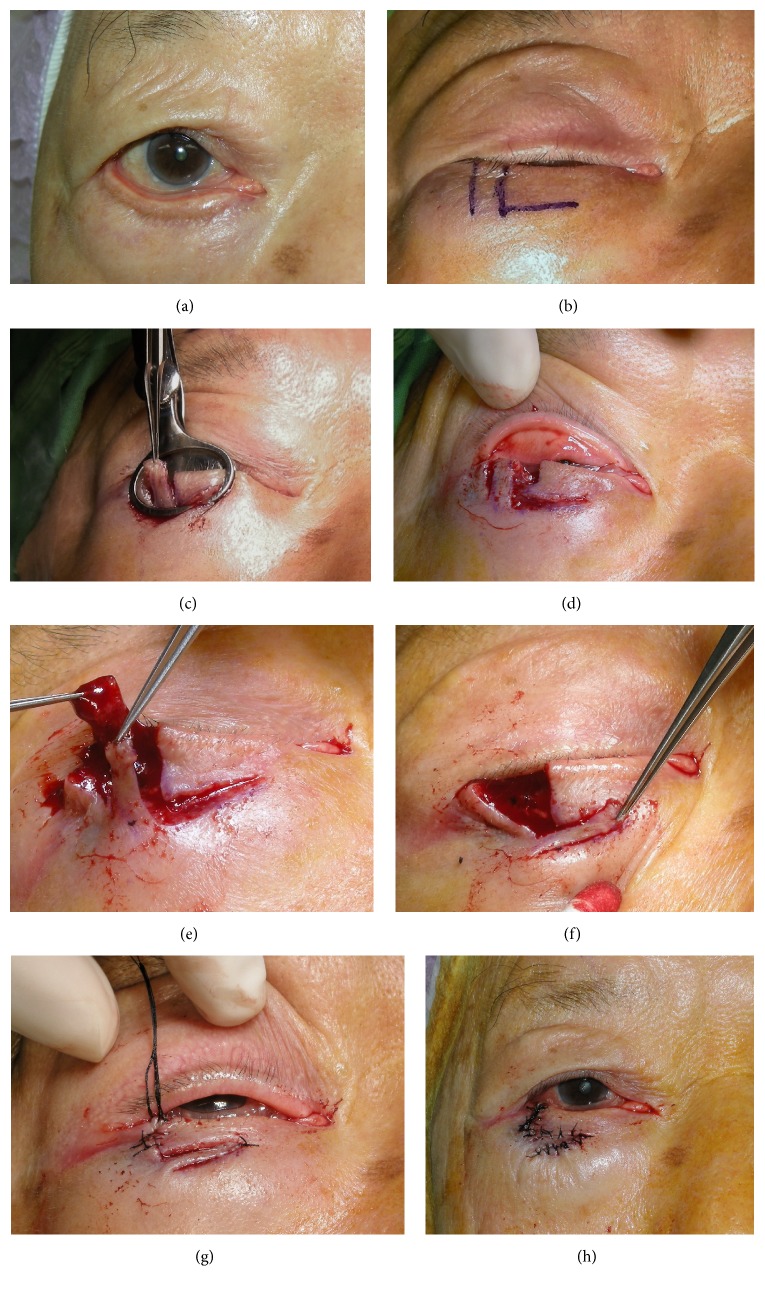
(a) Cicatricial ectropion of the right lower eyelid in an 83-year-old man (case 1). (b) Two vertical lines (12 to 15 mm) that were 8- to 12-mm wide were marked on the lateral third of the lower eyelid. The distance depended on the severity of the anterior lamella deficiency and on horizontal lid laxity. A 15- to 18-mm line was drawn medially and slightly upward along the base of the vertical flap. (c) After a lid plate or guard was inserted, two parallel vertical incisions were made using a number 15 scalpel blade. (d) A partial-thickness, horizontal relaxing incision was made medially along the base of the vertical pedicle. With Stevens scissors, the dissection was extended inferiorly to release any scar contracture of the lower lid. (e) and (f) A vertical myocutaneous flap was created by removing the posterior lamella and the lid margin (cilia portion) of the vertical pedicle and then rotated horizontally and sutured with 6-0 silk. (g) The lid margins of the two vertical incisions were approximated with three stitches of 6-0 silk, and the tarsus plate and preseptal and pretarsal orbicularis were sutured with 6-0 catgut. (h) The vertical myocutaneous flap was rotated horizontally and sutured with 6-0 silk.

**Figure 2:**
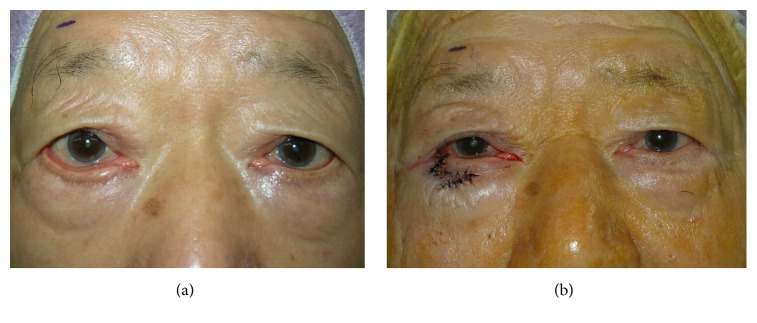
Photographs of case 1 taken before (a) and after (b) the operation.

1. Skin marking was used for cases with cicatricial ectropion (see case 1, Figures 1(a) and 1(b)). The length of the vertical cut was about 12 to 15 mm, and the width was about 8 to 12 mm, depending on the severity of the cicatricial ectropion and lid laxity. A 15- to 18-mm line was drawn horizontally and medially from the base of the vertical line.
2. After a lid plate or guard was inserted, two parallel vertical incisions were made using a number 15 scalpel blade through all layers of eyelid at the lateral third of the lower lid (Figure 1(c)).
3. A partial-thickness, horizontal, relaxing incision was made medially and slightly upward along the base of the prior vertical flap (Figure 1(d)). Then the Stevens scissors were used to spread in the quadrant to remove any scar contracture between the lower eyelid and upper face.
4. The anterior and posterior lamellae of the vertical flap pedicle were dissected and separated (Figure 1(e)). The vertical myocutaneous flap was created by removing the posterior lamella and lid margin (the cilia portion) of the vertical flap pedicle and was rotated horizontally and sutured with 6-0 silk (Figure 1(f)).
5. The lid margin of the two vertical incisions was approximated with three stitches of 6-0 silk, and the tarsus plate and preseptal orbicularis were sutured with 6-0 catgut. The vertical skin wound was closed with 6-0 silk (Figure 1(g)). The vertical myocutaneous flap was rotated horizontally and sutured with 6-0 silk (Figures 1(g) and 1(h)).
6. The long ends of the three previously tied 6-0 silk margin sutures were brought inferiorly and secured in that position by incorporating them into the last skin sutures (Figure 1(h)).  Figure 2 shows preoperative and postoperative images of this patient.

## 3. Results

**Figure 3:**
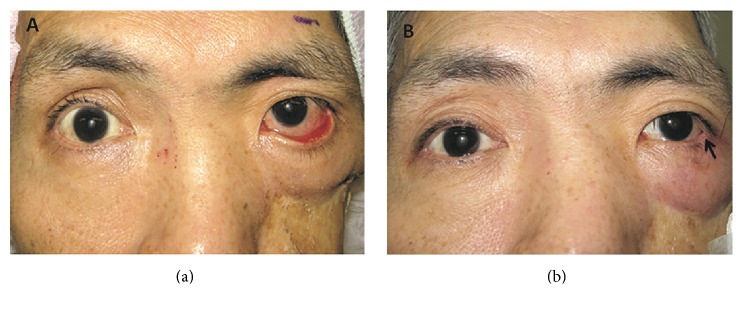
Case 2 was a patient with left maxillary squamous cell carcinoma subsequent to total maxillectomy. The ectropion was partially due to cicatricial contracture and partially due to a paralytic component (a). Additional lateral tarsorrhaphy (arrowhead in (b)) was performed for this patient (the photo was taken 6 weeks after surgery).

The study included a total of 20 eyelids in 20 patients who underwent the V-to-H rotational myocutaneous flap procedure (18 men and 2 women). Their mean age was 75.2 years (range: 41–90 years). The causes of cicatricial entropion were as follows: secondary to postsurgical scar (*n* = 13), Steven–Johnson syndrome (*n* = 2), longstanding antiglaucoma drops usage (*n* = 2), chemical burn (*n* = 1), postradiotherapy (*n* = 1), and involutional change in the eyelid (*n* = 1). Adjunctive lateral tarsorrhaphy was performed in 2 cases with combined cicatricial entropion and paralytic lagophthalmos (Figure 3).

**Figure 4:**
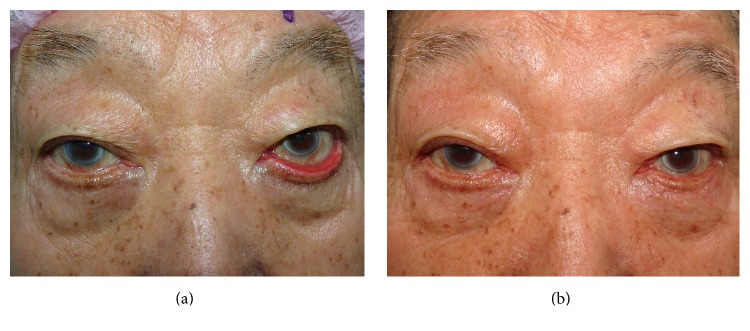
Photograph of case 3 before (a) and 4 months after (b) the operation.

At follow-up, all of the patients had good anatomical and functional improvement, satisfactory cosmetic results, and a decreased dependence on topical lubricants. Notably, the mean number of topical lubricant medications per patient significantly decreased from 1.4 to 0.15 (*P* < 0.001). None of the patients required further surgery. All flaps were viable, and the donor site scars were inconspicuous (Figure 4). No complications or recurrence was noted during the mean follow-up period of 33.2 months (range: 6–79 months).

## 4. Discussion

Patients with cicatricial ectropion who do not improve with conservative treatment often require surgical intervention to prevent vision-threatening corneal exposure. Full-thickness skin grafts or local transposition flaps are commonly used to lengthen the anterior lamella [4–8]. Donor site morbidity remains a concern with both surgical techniques. Moreover, often elderly patients with cicatricial ectropion also have horizontal eyelid laxity due to involutional changes, and both of these need to be corrected to achieve a better outcome. In addition, any downward traction during the healing process of the graft or flap may exacerbate the ectropion caused by horizontal eyelid laxity. Lateral tarsal strip or canthopexy in combination with local transposition flaps from the upper eyelid may help treat horizontal laxity [6–8, 12, 13]. However, care should be taken to ensure that there is enough skin in the donor site (upper eyelid) for adequate eyelid closure.

To address these issues, we used a local V-to-H rotational myocutaneous flap from the conventional wedge procedure in the lower eyelid to repair cicatricial lower lid ectropion. The local V-to-H rotational myocutaneous flaps have several advantages, including less donor site morbidity, similar skin color and texture, and correction of horizontal lid laxity at the same time. Some complicated cases with both cicatricial and paralytic ectropion with lagophthalmos can be challenging for plastic surgeons. We combined the V-to-H rotational myocutaneous flap procedure and adjunctive lateral tarsorrhaphy for these complicated cases and achieved good functional and cosmetic results (Figure 3).

**Figure 5:**
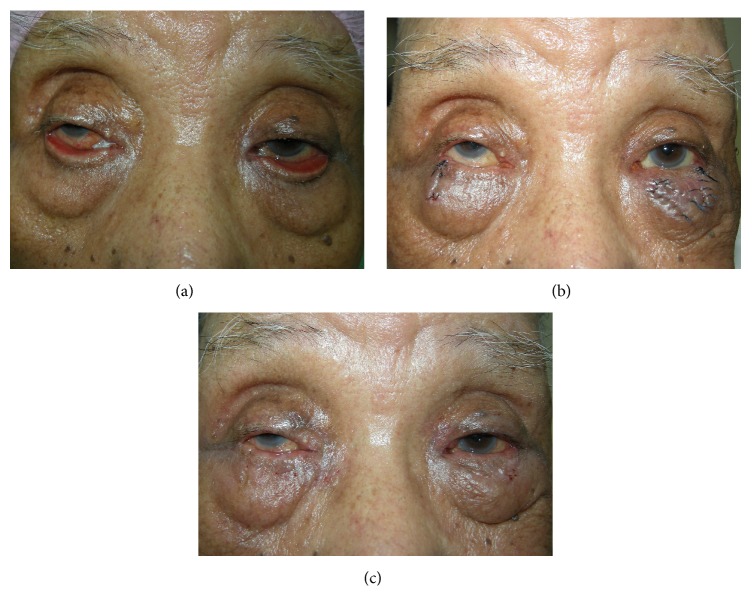
Case 4 had bilateral lower eyelid cicatricial ectropion (a) and simultaneously underwent pentagonal wedge excision in the right lower eyelid and the V-to-H rotational myocutaneous flap procedure in the left lower eyelid (b). The photograph was taken 1 year after the operation; there was marked improvement in the left lower lid that underwent the V-to-H rotational myocutaneous flap procedure, and there was residual lower lid scleral show in the right lower eyelid that was only treated with pentagonal wedge excision (c).

Figure 5 shows a patient with bilateral lower eyelid cicatricial ectropion who simultaneously underwent pentagonal wedge excision in the right lower eyelid and the V-to-H rotational myocutaneous flap procedure in the left lower eyelid. The postoperative result showed that the V-to-H rotational myocutaneous flap procedure provided additional benefits for the relief of anterior lamella shortening compared with pentagonal wedge excision alone (Figure 5).

In conclusion, our results suggest that the V-to-H rotational myocutaneous flap procedure is a good treatment option for correcting mild to moderate cicatricial lower eyelid ectropion, especially for elderly patients with horizontal eyelid laxity. Not only does the myocutaneous flap from the lower lid have similar skin color and texture, and less donor site morbidity, but also it achieved concurrent vertical lengthening and horizontal shortening of the lower eyelid to reduce the incidence of recurrent cicatricial ectropion.

## Figures and Tables

**Table 1:** Patients' characteristics and clinical data.

Case number	Age	Gender (M/F)	Laterality (OD/OS)	Cause	Number of topical lubricants before surgery	Number of topical lubricants after surgery	Follow-up (months)
(1)	83	M	OD	Involutional change	1	0	6
(2)^*∗*^	55	M	OS	Maxilla tumor s/p excision	2	0	64
(3)	90	M	OS	Antiglaucoma drops usage	2	0	6
(4)	84	M	OS	Antiglaucoma drops usage	1	0	6
(5)	75	M	OD	Lid tumor s/p excision	1	0	22
(6)	60	M	OS	Chemical burn	1	0	72
(7)	87	F	OS	Cheek tumor s/p excision	2	0	73
(8)^*∗*^	60	M	OD	NPC s/p radiotherapy	2	0	79
(9)	69	M	OS	s/p lower lid blepharoplasty	2	0	52
(10)	86	M	OS	Cheek tumor s/p excision	1	1	54
(11)	70	F	OS	Lid tumor s/p excision	1	0	6
(12)	41	M	OD	Lid laceration s/p repair	1	0	53
(13)	77	M	OS	s/p lower lid blepharoplasty	1	0	29
(14)	82	M	OD	s/p lower lid blepharoplasty	2	1	32
(15)	85	M	OD	Lid tumor s/p excision	1	0	20
(16)	86	M	OD	s/p lower lid blepharoplasty	1	0	31
(17)	75	M	OD	Steven Johnson syndrome	2	0	12
(18)	89	M	OD	Lid tumor s/p excision	1	0	6
(19)	75	M	OD	s/p lower lid blepharoplasty	2	1	14
(20)	75	M	OS	Steven Johnson syndrome	1	0	27

^*∗*^Patients with combined cicatricial and paralytic ectropion. M/F: male/female; OD/OS: right eye/left eye; s/p: status post; NPC: nasopharyngeal cancer.
